# Application of Metabolic Reprogramming to Cancer Imaging and Diagnosis

**DOI:** 10.3390/ijms232415831

**Published:** 2022-12-13

**Authors:** Yi-Fang Yang, Chien-Hsiu Li, Huei-Yu Cai, Bo-Syuan Lin, Cheorl-Ho Kim, Yu-Chan Chang

**Affiliations:** 1 Department of Medical Education and Research, Kaohsiung Veterans General Hospital, Kaohsiung 81362, Taiwan; 2 Genomics Research Center, Academia Sinica, Taipei 115, Taiwan; 3 Department of Biomedicine Imaging and Radiological Science, National Yang Ming Chiao Tung University, Taipei 11121, Taiwan; 4 Department of Biological Sciences, College of Science, Sungkyunkwan University, Seoburo 2066, Suwon 16419, Republic of Korea; 5 Samsung Advanced Institute of Health Science and Technology (SAIHST), Sungkyunkwan University, Seoul 06351, Republic of Korea

**Keywords:** cancer metabolism, metabolic reprogramming, molecular imaging, cellular uptake

## Abstract

Cellular metabolism governs the signaling that supports physiological mechanisms and homeostasis in an individual, including neuronal transmission, wound healing, and circadian clock manipulation. Various factors have been linked to abnormal metabolic reprogramming, including gene mutations, epigenetic modifications, altered protein epitopes, and their involvement in the development of disease, including cancer. The presence of multiple distinct hallmarks and the resulting cellular reprogramming process have gradually revealed that these metabolism-related molecules may be able to be used to track or prevent the progression of cancer. Consequently, translational medicines have been developed using metabolic substrates, precursors, and other products depending on their biochemical mechanism of action. It is important to note that these metabolic analogs can also be used for imaging and therapeutic purposes in addition to competing for metabolic functions. In particular, due to their isotopic labeling, these compounds may also be used to localize and visualize tumor cells after uptake. In this review, the current development status, applicability, and limitations of compounds targeting metabolic reprogramming are described, as well as the imaging platforms that are most suitable for each compound and the types of cancer to which they are most appropriate.

## 1. Introduction

Carbohydrates, lipids, nucleic acids, and proteins are the four main components required to maintain cellular function, and these are derived from metabolism. Apart from biosynthetic events that are primarily concerned with glucose, glutamine [1], fatty acids [2], and amino acids [3], the metabolism also involves rare vitamins [4], nucleic acids, and neurotransmitters (dopamine, serotonin, epinephrine, etc.). The de novo synthesis and salvage pathway of complex metabolic intermediates or metabolites facilitates the adaptation of cells to a changing external environment at any time, as well as to constants such as body temperature, heartbeat, nerve conduction, and signal transduction of the animal body index [5]. To achieve the integrity and balance of various bodily functions, the rate, ratio, and inducing factors that determine the events constitute the most important key elements of homeostasis [6].

One interesting finding is that some systemic diseases and tumors are associated with metabolic reprogramming—a series of domino effects that results in reversible or irreversible metabolic changes and dysfunction of the cells [7]. This process is also known as metabolic reprogramming. The most frequently observed phenomena in cancer cells are changes in metabolic function. To adapt to environments with various oxygen pressures, cancer cells will change their glucose use strategy, while at the same time using alternative substances to maintain their sources of energy. These include glutamine, fatty acids, ketone bodies, and amino acids [8], thereby changing the ratio between NADP^+^ and NADPH to enable the production of related substances capable of killing cells and promoting malignancy [9]. It is noteworthy that, in the current research, various metabolic reprogramming processes are explored that are unique to tumor cells. Despite the fact that these differences are highly correlated with progression, they can be further applied to achieve accurate diagnosis, treatment, imaging, and prognosis [10].

This review discusses a variety of metabolism-related products that have been developed as imaging tools, combination therapy options, and molecular markers. The objectives of this work are to discuss the metabolic pathways to which these metabolites belong, and how biomedical imaging tools and imaging devices that can be applied in the treatment of diseases and cancers have been derived from them. Additionally, some series have entered preclinical trials or have been approved for routine therapeutic applications.

## 2. Imaging Platforms for Coimaging with Metabolites

### 2.1. Positron Emission Tomography (PET)

Positron emission tomography (PET) is a method currently used for medical diagnosis of cancer, heart disease, and neuropsychiatric diseases, and also plays an important role in cutting-edge medical fields such as gene therapy [11]. Positron tomography, as the name suggests, is a computed tomography examination based on the action of positrons and belongs to the field of nuclear medicine imaging diagnosis [12]. The public is familiar with PET. Unlike computed tomography (CT) or magnetic resonance imaging (MRI) scans, its imaging relies on the emission of positively charged electrons, known as positrons [13]. Isotopic drugs (positron drugs) are produced through a decay process. The principle of PET is that after the positron drug is intravenously injected into the human body, the positrons generated by the decay process travel less than 1 mm in the human tissue, because when they collide with the negatively charged electrons, they cancel out and destroy each other (in physics, this is called mutual destruction or annihilation). In PET, high-energy gamma rays of 511 KeV (Kilo Electron Volts) are emitted at 180 degrees during the annihilation of positrons and electrons. A positron tomography scanner (that is, an instrument that performs PET, also called a PET scanner) detects these pairs simultaneously and uses a computer to reconstruct an image of the distribution of positron-emitting isotopes within the tissue or organ [14].

Drugs used in PET mainly include several positron-emitting radioactive isotopes: oxygen-15 (^15^O), nitrogen-13 (^13^N), carbon-11 (^11^C), and fluorine-18 (^18^F); these isotopes can synthesize many metabolic molecules that exist in or are needed by the body, such as glucose, amino acids, etc., and can be used in research to explore normal or pathological metabolic functions of the human body [15]. The most commonly used positron isotope drugs currently in clinical use are [^18^F]-deoxyglucose ([^18^F] 2-fluoro-2-deoxy-D-glucose, also called FDG). The four short-half-life positron isotopes mentioned above are produced by a cyclotron. Because of the short half-life, positron tomography equipment is usually installed close to the cyclotron, and this is convenient for obtaining medicines for examination.

The key clinical value of the PET test is that it is an approach for the early diagnosis of cancer in current medical technology. It detects primary tumors of metastatic cancer cells, cancer staining, and enables correct evaluation prior to cancer surgery (for example, whether surgery is required, and can be performed, or whether direct chemotherapy or radiation therapy is more suitable for the patient), as well as assessment and follow-up of postoperative results. Similarly, it allows correct assessment prior to chemoradiotherapy and assessment and follow-up of post-treatment effects. Preparations currently available for tumor positron tomography include FDG, fluorine-18-labeled thymidine (called FLT) or choline, carbon-11-labeled acetate, and methionine. To distinguish benign from malignant tumors, the examination mainly focuses on the difference in the absorption and retention of deoxyglucose between malignant tumors and normal tissues (generally, malignant cells can absorb more glucose and remain in cells longer). With regard to FDG injectors, most malignancies show a high uptake and positron tomography can be detected in vitro using positron imaging equipment. If lesions with abnormally high absorption are observed, there is a possibility of malignancy [16]. Various malignancies can be diagnosed and treated with FDG, in particular colorectal, esophageal, lymphoma, head and neck, melanoma, and thyroid cancers. Other positron drugs such as flucytosine (used for imaging cancers, including brain tumors), choline fluoride (used for imaging other cancers of the urinary system), and normalized carbon 11 acetate or methionine can also be used for imaging different types of tumor, and their clinical value and benefits require further exploration and evaluation [17]. Furthermore, positron tomography can use estrogen or progesterone derivatives in preoperative angiographic evaluations. Assessing estrogen or progesterone receptors in breast cancer tissue is also equivalent to assessing the effect of hormone therapy on breast cancer [18].

In the metabolic activity of the myocardium in a normal heart (especially in a state of starvation), free fatty acids (FFA) are the most important source of energy, with long-chain fatty acids providing about 70% of the energy required. Although carbohydrates provide only 30% of the energy required by the myocardium, blood glucose and insulin concentrations increase after eating, and free fatty acid concentrations increase. Currently, the main source of myocardial energy is glucose [19]. When myocardial hypoxia or insulin is elevated as a result of myocardial pathological conditions, free fatty acids in the granular glands and the myocardium immediately increase glucose levels by increasing glycolysis. Therefore, the metabolic activity of the myocardium can be adjusted at any time according to physiological and pathological conditions. Positron tomography reveals myocardial matrix use and metabolic activity, whereas cardiac positron imaging to assess FDG is the main positron reagent for evaluation of myocardial metabolic activity. Its main clinical application is for the evaluation of cardiac activity after myocardial infarction. A viable myocardium in the infarcted area helps the cardiologist select the most appropriate treatment strategy for the patient. PET scans include cardiac perfusion (^13^N-NH_3_PET) and metabolic examination (FDG PET). The cardiac perfusion ^13^N-NH_3_PET scan can detect abnormal perfusion, which indicates a decrease in regional myocardial blood flow. The phenomenon of decreased metabolism revealed by the FDG examination indicates that this local part of the heart muscle is necrotic and scarred, and even balloon dilation or coronary bypass surgery will not improve heart function. If the FDG examination finds areas of reduced blood perfusion, there will still be some, or even increased metabolic activity, suggesting myocardial ischemia. With oxygen, heart muscle cells are alive, and dilation or bypass surgery can improve heart function.

### 2.2. Magnetic Resonance Imaging (MRI)

A standard neuroimaging technique for the diagnosis of brain tumors is magnetic resonance imaging (MRI), which uses T1 or T2 fluid-attenuated inversion recovery (FLAIR) and gadolinium-enhanced T1-weighted images [20,21]. When radiation or chemotherapy has been applied to the area prior to the magnetic resonance imaging, the results are excellent, but the specificity is limited. In T1-weighted sequences (T1w), contrast-enhancing components can quantify the disruption volume of the blood–brain barrier (BBB), allowing for the detection of malignant tumors. T2-FLAIR-weighted images demonstrate abnormalities resulting from a combination of non-enhancing tumors, subfocal edema, and treatment-related changes (gliosis, leukoencephalopathy, and necrosis) [22,23]. Despite this, this technique has several limitations. Specifically, when using conventional MRI during the follow-up phase, it is difficult to distinguish between pseudoprogression after treatment completion and tumor progression. The reason for this is that contrast enhancement on magnetic resonance imaging indicates a leakage through the blood–brain barrier of the contrast medium, which may be related to the treatment being administered [24].

It is possible to distinguish pseudo- and real progression more effectively using advanced MR imaging techniques, such as diffusion- and perfusion-weighted MRIs, as well as magnetic resonance spectroscopy, compared to anatomical MR imaging alone [25]. However, with MRI, the quality of the imaging depends on the quality and structure of the object being inspected. The current diagnosis for imaging is to combine the above-mentioned PET and CT to improve accuracy and degree [26]. In brain regions in particular, if magnetic resonance can be combined with specific metabolically relevant radiotracers, not only can high positive correlations be observed, but this will also inform the grading and characterization of brain tumors and the prediction and assessment of treatment response [27].

## 3. Radiotracers Available for Cancer and Disease

**Figure 1:**
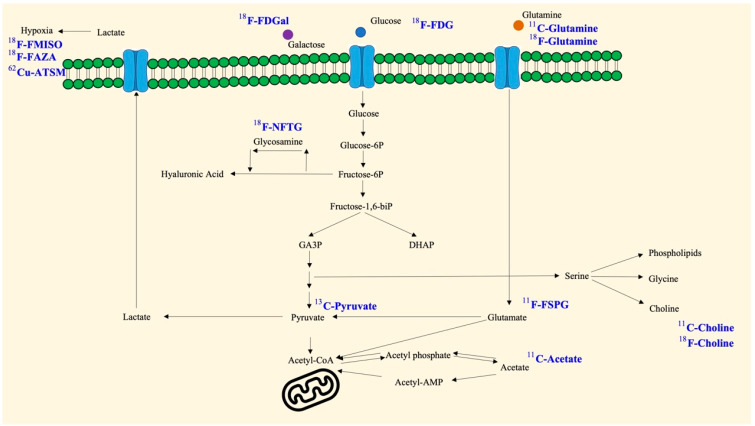
Available metabolism-based radiotracers and their background. The schematic model shows that monosaccharides (glucose and galactose) and glutamine can be taken up by cells, followed by cascade catalysis and synthesis. Glucosamine, pyruvate, glutamate, acetate, and choline may be intermediates or have further consequences. Furthermore, hypoxia is a metabolic reprogramming event with corresponding detectable targets. The blue color represents the derived radiotracer products.

**Figure 2:**
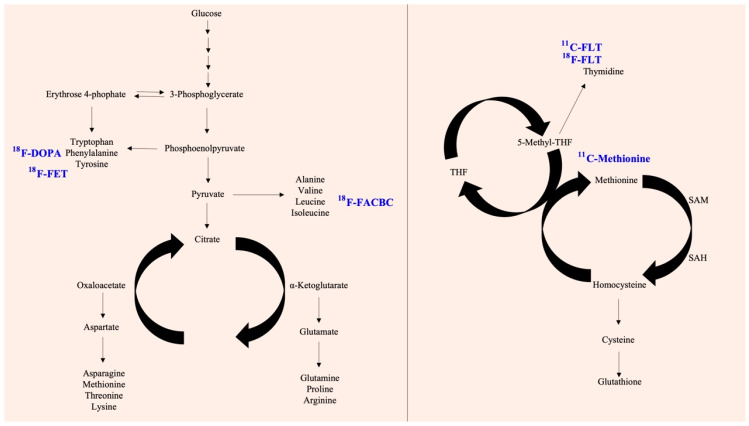
Available metabolism-based radiotracers and their background. The schematic model shows glycolysis and the further TCA cycle, amino acid biosynthesis. In the path, there are some potential targets with radiotracers (**left panel**). In addition, the coupling of the folate cycle to the methionine cycle is shown (**right panel**). The blue color represents the derived radiotracer products.

As mentioned above, the microenvironment, immune system, and signaling pathways are abnormal in cancer and other diseases and have been shown to be closely related to metabolic events [28]. In particular, subpopulations of lesions themselves are particularly dependent on specific nutrients and increase the rate of absorption and generation. Because of this property, many radiotracers that have been developed based on metabolites can be used for visualization, localization, and quantification (Figure 1 and Figure 2).

### 3.1. Glucose

Glycolysis is a pathway that uses glucose for metabolism and catalysis, and there are many related studies and evidence. Scientists have found that there are many diseases and cancers associated with abnormal glycolysis. The unbalanced proliferation activity of tumor cells results in increased glucose uptake by tumor cells. This includes not only an increase in the glucose uptake rate, but also an increase in the efficiency of the transporter responsible for the membrane [29].

Several clinical studies, such as The Cancer Genome Atlas (TCGA) and Gene Expression Omnibus, have demonstrated that certain cancer types exhibit selective overexpression of glucose transporters (GLUTs). It was found in Kim’s research that the expression of GLUT2 was significantly higher in hepatocellular carcinomas than in normal tissues when compared with other submembers of the GLUT family. Accordingly, this difference is positively correlated with poor prognosis or clinical stage of patients, thus it may serve as a prognosis factor [30]. It has been found that GLUT3 is overexpressed in several carcinomas, including adenocarcinoma and glioblastoma [31,32,33].

The downstream genes responsible for the 10 steps of the glycolysis response will also follow this pattern by increasing their enzyme activity and expression levels [34], consequently activating several signaling transduction pathways and transcription factors. This process is encapsulated in the Warburg effect theory to accelerate glucose/lactate conversion to respond to oxygen concentrations in tumor cells [35].

In highly proliferating specific cells, there is a specific difference between glucose uptake and glycolysis rates. This has led to the development of molecules that target glucose for use as markers. Fluorodeoxyglucose (FDG) is an analog of a glucose molecule, similar in structure to glucose. FDG is taken up via the GLUT-1 transporter and phosphorylated to FDG-6-phosphate by hexokinase [36]. Because of the lack of C-2 hydroxyl groups, further metabolism is impeded, and the FDG-6-phosphate is trapped within cells, accumulates at high absorption, and is eventually metabolized. Many basic and clinical studies have used FDG as an inhibitor to interfere with the normal glycolysis reaction by selecting specific cells with high sugar uptake. Radiation scientists also use the abovementioned imaging equipment. Radiolabeling technology has been used to design products such as ^18^F-FDG, which is derived from PET and can track the FDG signal to locate the tumor location. The advantage of FDG-PET is that it can be used as an early assessment of whether the tumor metabolism has changed and to evaluate whether a change in treatment strategy is necessary [37]. As a matter of fact, this depends on the high glycolytic rate of the tumor or disease model, if the glycolytic rate is insufficient, there will be no significant difference in the degree of contrast in imaging [37,38].

There are many other modifications and improvements of FDG, including radioisotopes, peptides (linear and cyclic), and some conjugations [39]. Namavari et al. used ^18^F-FDG as a prosthetic set to design ^18^F-FDG-RGD and ^18^F-FDG-cyclo (RGDDYK) by one-step radiosynthesis. This design can increase the binding affinity to the integrin α_v_β_3_. The imaging of integrin αvβ3 expression in vivo is a potentially useful method for diagnosing tumors and their metastases to provide a better understanding of tumor angiogenesis and monitor the effects of target-specific antiangiogenic therapy. Senisik et al. also describe a single-step, high-efficiency conjugation of glycylglycine (GlyGly), which is a small peptide, with [^18^F] FDG, without the need for purification. Researchers without cyclotron facilities can easily perform efficient radiolabeling and use [^18^F] FDG-GlyGly to improve biodistribution studies [40].

Whole body scans are mainly used for tumor examination. It is recommended that the patient fast for a minimum of six hours before the test and maintain a blood sugar level less than 120 mg/dL. After intravenous injection of about 5 to 10 millicuries (mCi) of FDG, the subject must lie down and rest for 45 min, so that the FDG can accumulate in the tumor, be adequately eliminated from normal tissues, and pass through the kidneys and bladder [41]. After 45 min of excretion, after removal of urine, the patient lies down on the scanning table for examination. Generally, it takes about an hour to scan from the head to the upper third of the thigh [42]. To reduce the influence of body tissue on the attenuation effect of gamma rays, a ^68^Ge radiation source is generally used for another transmission scan to correct the tissue attenuation effect, and an additional 15 to 30 min of examination time is required. After scanning, tomographic image reconstruction and analysis can be performed [43]. If necessary, the absorption rate of FDG by the lesion, such as the standard uptake value (SUV), can be measured. There is the option for a local scan to be added, and the examination time for this is approximately 20 min, to allow for the differential diagnosis of benign and malignant lesions in specific organs (such as lung masses).

### 3.2. Pyruvate

In the glycolytic pathway, lactate and pyruvate act as two branches, leading to glycolysis and anaerobic lactic fermentation or aerobic oxidative phosphorylations (OXPHOs) [44]. The conversion of glucose to pyruvate occurs through glycolysis in cancer, whereas lactate is produced by lactate dehydrogenase (LDH) [45]. In this manner, cancer cells are exploited for their unique properties. It has been reported that some studies have attempted to increase detection sensitivity in solution-state nuclear magnetic resonance experiments by using dynamic nuclear polarization of ^13^C-labeled pyruvate [46]. In vivo imaging of the spatial distribution and metabolism of a labeled molecule following intravenous injection in mouse models is possible by using ^13^C magnetic resonance spectroscopic imaging (MRSI). In a reaction catalyzed by LDH, the decrease in label flux between hyperpolarized [1-^13^C]pyruvate and lactate can be measured to determine whether mouse lymphomas are responding to drug treatment. There may be several reasons for the reduction in flux, such as loss of cell enzymes, reduced tumor cellularity, or reduced concentrations of lactate and NAD(H) in the tumor. Therefore, it is important to compare these results with FDG-PET to improve the value of FDG-PET in its current board-certified clinical application [47].

### 3.3. Galactose

A variety of cancers have been successfully treated using the combination of ^11^C-acetate and FDG PET/CT. However, the detection sensitivity of HCC types for the small primary subtype is low [48]. Several studies have demonstrated that HCC has a greater effect on food intake and galactose production within the body. The expression of galactose metabolism related enzymes such as galactokinase (GALK1) and galactose-1 phosphate uridylyltransferase (GALT) in HCC is higher than that of normal liver tissues and is critical for HCC proliferation [49]. It has been demonstrated that galactose is metabolized specifically in hepatocytes, and thus a proof-of-concept imaging test has been developed for the diagnosis of liver cancer [50]. Compared with other tracers, 2-deoxy-2-[^18^F]fluoro-D-galactose ([^18^F]FDGal) was found to accumulate in the liver more frequently [51,52]. FDGal can be synthesized using commercially available FDG production kits with only minor modifications, enabling this process to be implemented at most clinical PET facilities [53]. ^18^F-FDGal PET/CT was able to detect previously unknown extrahepatic diseases and regions when used in conjunction with contrast enhancement CT (ce-CT) for liver lesions. [50]. Despite this, the ^18^F-FDGal PET/CT images are still limited by the extremely high uptake of ^18^F-FDGal in normal liver tissue as well as by the heterogeneity of uptake in cirrhosis patients. A comparison and discussion of three ^18^F-FDGal PET protocols was conducted by Horsager et al., in order to achieve the highest tumor/background ratio and optimal observations [54]. Furthermore, some studies have noted that coadministration does not improve the interpretation, detection, or statistical values of ^18^F-FDGal PET/CT images [55].

### 3.4. Choline

A nutrient that builds cell membranes and phospholipids, choline, is necessary for brain development and memory, and is a precursor to acetyl-CoA, which is involved in muscle control and neuronal transmission. The activity of choline metabolism-related metabolic enzymes is abnormally increased in cancer and is related to malignancy. Activated phosphatidylcholine-specific phospholipase D (PC-PLD) and phospholipase C (PC-PLC) are associated with abnormal tumor proliferation in ovarian cancer [56]. Increased levels of glycerophosphorylcholine (GPC) and phosphorylcholine (PC) promote epithelial-to-mesenchymal transition (EMT) in ovarian cancer [57]. In some studies, highly saturated phosphatidylcholine species, CDP-choline species, and phosphocholine species are significantly higher in HCC tumors than in adjacent liver and intrahepatic cholangiocarcinoma (ICC) tumors [58].

Fluorine (18F) has been labeled for PET and CT to detect chronic liver diseases and liver cancers [59]. Despite having a similar function to ^18^F-FDGal, ^18^F-fluorocholine uptake shows significant associations with liver uptake and neuroinflammatory and fibrotic changes found in chronic liver disease patients’ histology [59].

Choline can also be labeled with carbon-11 (^11^C) as a positron emitter for molecular imaging [60]. Among several short-lived positron-emitting radionuclides used in PET imaging, ^11^C has a unique probability (t1/20.4 min, *E*_β+_ = 1.98 MeV) that is easily distinguished from the nonradioactive ^12^C molecule. In addition, ^11^C has a short half-life (20.33 min) and most ^11^C will decay into ^11^B through β decay, which is less harmful to the human body and has increased applicability [61]. Evaluation of *N*-methyl-[^11^C]choline in PET in patients with recurrent prostate cancer and localized parathyroid adenomas that exhibit biochemical recurrence has been performed. It has been evidenced that the choline kinase alpha (XKα) overexpression can be found in primary hyperparathyroidism, parathyroid hyperplasia, and neoplastic lesions [62]. Liu et al. evaluated the value of ^11^C-choline PET in patients with primary hyperparathyroidism and indicated that this tool had potential for the location of parathyroid adenomas when ultrasound and ^99m^Tc-sestamibi imaging yielded negative or discordant results [63]. Although the study by Noltes et al. was a retrospective analysis, the results were still promising, showing high accuracy and sensitivity [64]. Furthermore, ^11^C-choline PET can also be applied to prostate cancer. Although serum prostate specific antigen (PSA) testing is often used to monitor disease recurrence after definitive therapy for prostate cancer [65], Krause et al. identified a positive correlation between ^11^C-choline PET detection rate and serum PSA levels [66]. Several clinicopathological factors of prostate cancer can be statistically significant for ^11^C-choline uptake, even when the PSA value and kinetics are low [67]. Despite some evidence, ^18^F-FDG is rarely used in the evaluation of prostate cancer, because ^18^F-FDG PET has a low sensitivity for the detection of prostate cancer, as exemplified by the fact that its sensitivity for detection of bone metastases is lower than that of bone scintigraphy [68]. This also increases the application value of ^18^F/^13^C-choline.

### 3.5. Acetate

As with lactate, pyruvate can be converted into acetate through keto acid dehydrogenase. Acetate can serve as an intermediate product of shuttle metabolism in cancer cells that interacts with surrounding cells or is capable of resisting external stress [69]. Studies have shown that cancer cells are capable of adapting to low oxygen environments through the conversion of acetate. Specifically, mitochondria-localized acetyl-CoA synthetases (ACSS 1/2) convert acetate into acetyl-CoA for use in the TCA cycle or fatty acid synthesis [70]. The abnormal expression of ACSS2 has also been observed in several cancers, making it a potential target for cancer therapy [71,72,73]. Acetate, in addition to serving as a buffer for metabolic intermediates, can also play the function of compensating for acidic PH environments through its role in histone acetylation [70].

Similar to choline, acetate is a precursor for lipid biosynthesis [74]. Studies have shown that it is an important bioenergetic fuel for tumor cells. It has been claimed to promote histone acetylation and other epigenetic modifications. Despite the lower circulating concentrations of acetate, it can still surround the intracellular circulation and tumor microenvironment [70]. The difference in kinetics between normal myocardium, normal renal parenchyma, and renal cancer tissue has been proven. Myocardial and normal renal tissue showed rapid washing, consistent with the predominant oxidation to CO_2_ through the tricarboxylic acid cycle. In contrast, the tracer was retained in tumor tissue, probably because of the use of ^11^C-acetate as an important substrate for the generation of membrane lipids [75]. Through comparison of multiple organs and conditions using imaging tools, acetate has been verified as suitable for the detection of prostate cancer.

For prostate cancer, ^11^C-acetate can be used as a predictive, prognostic, and intermediated endpoint biomarker. ^11^C-acetate can be detected under low PSA conditions, but it is hard to differentiate between benign prostatic hyperplasia and prostate cancer. In contrast, ^11^C-acetate PET can be used to assess the regional lymph node and recurrence status [76]. As mentioned previously, the sensitivity of the diagnostic range of ^11^C-acetate and choline PET probes is important in the evaluation of prostate cancer. Although ^11^C-acetate has the advantage of low urinary excretion, ^18^F-choline is more widely used because of its longer physical half-life. ^11^C-acetate and ^11^C-choline can only identify about 50% of recurrence sites, although they both have limitations in patients with PSA levels below 1ng/mL [77].

### 3.6. Pivalic Acid

In some clinical studies, glucose is not a suitable tracer, because cells can also produce energy through other metabolic pathways, such as fatty acid oxidation. Some de novo biosynthesis or short-chain fatty acid salvage pathways are important sources of nutrients for cell growth and proliferation [78]. These include acetate and ^18^F-fluoropivalate (3-^18^F-fluoro-2,2-dimethylpropionic acid, ^18^F-FPIA). Unlike acetate, pivalate cannot be oxidized to carbon dioxide in mammalian cells because it carries a *tert*-butyl substituent [79]. Pivalate is esterified in vivo in normal tissue, and the resulting ester enters plasma and is absorbed by cells inhibited by L-carnitine or rapidly eliminated in urine [80]. Dubash et al. evaluated the safety, biodistribution, and internal radiation dosimetry profile of ^18^F-FPIA in healthy volunteers [81]. A current phase 2 clinical trial (NCT04717674) has enrolled some solid tumor patients who are undergoing ^18^F-FIPA PET imaging. Their ^18^F-FIPA tumor uptake will be calculated, and several histological markers of tumor metabolism measured. These biomarkers include Ki-67 and typical enzymes related to fatty acids (SDHA, CPT1/CAT1, CACT, SLC22A2, SLC22A5, and SLC25A20).

### 3.7. Cyclobutanecarboxylic Acid

Fluoro-18 (^18^F) fluciclovine (anti-1-amino-3–^18^F-fluorocyclobutane-1-carboxylic acid [FACBC]) is a radiolabeled amino acid analog that is upregulated by amino acid transport in a variety of cancer cells, and prostate cancer in particular [82,83,84]. With its low radioactivity background, it is effective for detecting prostate progression, even in cases of lymph node or bone metastasis below 5 mm [85].

It can be taken up through the human L-type amino acid transporter and alanine-serin-cysteine transporter systems [86]. By the same principle, FACBC is analogous to FDG in that it is a metabolite that, when taken up by cells, cannot be metabolized and remains in the tumor area. The sample application principle also includes ^18^FDG, FACBC, and ^11^C-choline. They each use glucose transporter (GLUTs), L-type amino acid transporter (LAT/ASCT2), and choline transporter, respectively. The ^68^Ga PSMA-11 and ^111^In-Capromab pendetides target the specific antigen PSMA [87].

### 3.8. Methionine

Methionine is a natural essential amino acid involved in various types of biosynthesis. It is possible for methionine cycle biosynthesis to convert methionine into S-adenosylmethionine (SAM) in order for methyl donors to be synthesized for genetic processes through methionine adenosyltransferase (MAT) [88,89]. The Hoffman effect is a phenomenon that describes proliferating cells relying on methionine for growth [90]. Methionine has been found to be present in high amounts in certain cancer cells [91]. Compared with normal cells, cancer cells deprived of exogenous methionine in the environment grow and move more slowly [92]. Consequently, labeling methionine is considered an effective method for identifying cancer cells. For use as a radiotracer, methionine is labeled using carbon-11 and its distribution is usually detected using PET. MET images of brain tumors often show increased uptake in tumors with the presence of amino acid transporters (sodium-independent L-transporters, LAT1, 2, and 3), MET metabolism, tumor vascular bed-dependent blood flow, microvessel density, and blood–brain barrier distribution. This property has made ^11^C-Met a popular imaging agent in brain cancer patients [93]. The conditions to which it has been applied range from genetic alterations and relapse status to post-chemotherapy evaluations [94,95,96,97,98,99].

Park et al. also described the application of ^11^C-Met in intracranial germinoma, observing that ^11^C-Met PET parameters had a significant association with tumor location, sex, *KRAS* variant, and symptoms [100]. Morales-Lozano et al. also compared ^11^C-Met and ^18^F-FDG in multiple myeloma, and they confirmed that ^11^C-Met PET/CT was more sensitive than ^18^F-FDG for evaluation of myeloma tumors [101]. Currently, a clinical trial is underway to evaluate the response of a more comprehensive range of cancer types (NCT00840047).

### 3.9. Glutamine

Glutamine is the most abundant and versatile amino acid in the body. Many metabolic events are expected to be associated with glutamine use. Even in immune cells, its frequency of use approaches or exceeds that of glucose. In energy metabolism, glutamine is a supplementary pathway other than glucose and can be involved in the TCA cycle and events within the mitochondria. Glutamine is an essential nutrient for lymphocyte proliferation and cytokine production, macrophage phagocytosis and secretory activities, as well as neutrophil bacterial killing [102].

As well as its role as an energy source, glutamine also plays an important role in maintaining a balance between the pressures produced by antioxidants and oxidative stress. Meanwhile, it has been found that Glutamine is reliant on Methionine in some cancer cells [103]. This phenomenon is especially evident in clear cell renal cell carcinomas and breast cancer, in which both tumor microenvironment cells and tumor cells will deplete glutamine levels. This depletion limits the harmful effects of peripheral immune cells on cancer [104,105]. As a result, targeting Glutamine selectively is an effective method of diagnosis.

To evaluate the safety and tumor imaging characteristics of fluorine 18-(*2S,4R*)-4-gluoroglutamine (FGln), the Dunphy Group performed scans on cancer patients [106]. Cohen et al. reported that ^11^C-Gln could be applied to metastatic colorectal cancer. The status quo is that some targeted therapies are used in combination with glutamine metabolism inhibitors, but the study lacked appropriate diagnostic tools for assessment of early response. Therefore, calculated glutamine influx and glutamate efflux (^11^C-Gln and ^18^F-FSPG, respectively) have been recruited for further safety and biodistribution evaluation (NCT03263429) [107]. Glioma study indicates that although ^18^F-FGln is not superior to many current neuroimaging modalities, it may provide complementary biological information specifically about metabolic nutrient uptake relevant to glioma pathology [108]. ^18^F-FGln uptake in gliomas is positively correlated with glioma progression, and can be indicative of gene alterations (*PTEN* or *IDH1*) events or response to chemotherapy or radiation therapy. More recently, the glutamine-derived development, ^18^F-labeled glutamate (4*S*)-4-(3-^18^F-fluoropropyl)-1-glutamate, has been considered a PET tracer in preclinical models and human subjects [109]. However, the disadvantages of ^18^F-FGln still include neuroinflammation or disruption of the BBB that cannot be clearly observed [108].

### 3.10. Fluoropropyl-L-Glutamic Acid (FSPG)

Glutamic acid and glutamine are both interconvertible. Furthermore, *N*-acetylglutamate, α-ketoglutarate, 1-Pyrroline-5-carvoxylate, *N*-Formimino-L-glutamate, and *N*-acetylaspartylglutamic acid (NAAG) can produce glutamic acid through their corresponding enzymes [110]. Glutamic acid and glutamine form the glutamate metabolism that comprises several reversible and irreversible reactions.

FSPG could be applied to measure system XC-transporter activity, because it contributes to the important function of glutathione biosynthesis and the glutaminolytic pathway. These transporter families include SLC1A5, SLC38A1/A2, and SLC7A11 [111]. Unlike glycolysis, the glutaminolytic, glutathione biosynthesis, and redox balance pathways are dominant metabolic reprogramming events in cancer [112]. According to previous research, it can be detected and quantified in several types of cancer, including head and neck, colorectal, and non-Hodgkin lymphoma, and can play a complementary role in insignificant and ineffective types of ^18^FDG. There are many unknowns in the related research, but it has development potential for future systems.

### 3.11. L-Tyrosine

L-tyrosine arises from amino acid biosynthesis and is considered one of the necessary amino acids. In addition to being associated with Huntington’s disease and phenylketonuria, abnormal tyrosine metabolism has also been demonstrated to be associated with malignancy in several cancers [113]. It is evident from the method of blood detection that tyrosine levels will increase abnormally in HCC [114]. Based on the analysis of large sets of data such as TCGA and GEO, it appears there is a strong correlation between genes associated with tyrosine metabolism and poor prognosis in HCC [115]. A study conducted by Sun also suggests that acetyl-CoA generated by tyrosine metabolism may promote stemness activity in HCCs, thereby making them resistant to therapeutic treatment [116].

With amino acid PET, one can obtain information on the metabolism of tumor cells, which is complementary to structural imaging using magnetic resonance images [117]. Common amino acid-type radiotracers include ^18^F-FET and ^11^C-Met. Because ^18^F-FET has lower uptake by inflammatory cells than ^11^C-methionine or 18F-FDG, it is useful for differentiating tumors from treatment-induced necrosis [118]. Labeled with L-tyrosine fluoroine-18 fluorine, the approach with MRI can achieve 93% sensitivity and 94% specificity in glioma tissue [119].

In current glioblastoma treatments, temozolomide (TMZ) is considered the standard chemotherapy regimen for patients, but it leads to drug resistance and survival prolongation is limited. However, ^18^F-FET may be a valuable tool for predicting the outcome of therapy before the commencement of TMZ maintenance therapy [120]. Merging this evidence, ^18^F-FET has been identified as having a good diagnostic performance for the initial assessment of patients with new isolated brain lesions [121]. A previous retrospective study also claimed that a combination of static and kinetic ^18^F-FET parameters achieved a higher diagnostic accuracy than conventional MRI in distinguishing between recurrent or progressive disease and treatment-related changes [122]. 

### 3.12. Thymidine

Thymidine is the DNA nucleoside that pairs with deoxyadenosine in double-stranded DNA [123]. In cell biology, it is used to synchronize cells in the S phase, so its production is closely related to the cell cycle [124]. Cancer cells often exhibit abnormal cell proliferation, and many treatments have been developed to inhibit genetic replication. Inhibitors of thymidylate synthase (TS), such as 5-fluorouracil (5-FU), are available [125]. It has been demonstrated that combining 5-FU and leucovorin or oxaliplatin significantly increases the chance of TS and improves outcomes, but it also has great side effects on patients with advanced CRC [126,127]. It is, therefore, beneficial to develop early detection of patients in order to increase the diagnosis rate and formulate further treatment strategies.

In addition to several common thymidine analogs (BrdU and EdU) that have proliferation monitors, the thymidine analog 3′-deoxy-3′[^18^F] fluorothymidine (FLT) has been developed as a proliferation marker for cancer research. Interestingly, the rate-limiting enzyme of FLT metabolism, the pyrimidine-metabolizing enzyme thymidine kinase-1 (TK-1), is overexpressed in pancreatic cancer cell lines and pancreatic cancer [128]. ^18^F-FLT is transported through the cell membrane and trapped inside the cell after phosphorylation by thymidine kinase 1 (TK1). Although ^18^F-FLT is not incorporated into DNA, it is still considered a surrogate marker of cellular proliferation because TK1 activity is closely regulated by the cell cycle [129]. A close relationship could be demonstrated between tumor retention and cell proliferation, suggesting that ^18^F-FLT is a promising marker for monitoring treatment response. Unlike ^18^F-FDG, ^18^F-FLT is not taken up by inflammatory cells, which could reduce the rate of false positive findings in both in pancreatitis and inflammatory tissue after therapy [128]. A comparative study revealed that ^18^F-FLT was more sensitive than ^18^F-FDG and ^18^F^-^fluorethylcholine in a human pancreatic xenograft model using SCID mice [128]. Similarly, TK-1 and DTYMK kinase and some related transporters, SLC28A1 and SLC29A3, increased significantly in the PDAC group [128]. The use of ^18^F-FLT is not limited to pancreatic cancer, but has some additional applications in different types and combinations. The Cieslak group has shown that pharmacological ascorbate (AscH^−^) induces cytotoxicity and oxidative stress in pancreatic cancer and then contributes to the radiosensitizer function monitored by ^18^F-FLT PET [130]. Collet et al. also claimed that ^18^F-fluoro-_L_-thymidine could provide an additional indicator for the staging of gliomas, in particular distinguishing between stage II or stage III (low-grade glioma) and stage IV (glioblastomas) [131]. Furthermore, there is an ongoing study to evaluate 18F-FLT uptake in non-small cell lung cancer following pemetrex treatment. Because of the response and activation of the dexamethasone/pemetrexed thymidine salvage pathway inhibited by dexamethasone/pemetrexed [132], the authors hypothesize that this strategy can be detected as an increase in FLT tumor uptake that subsequently decreases with reduced proliferation. In this study, the FLT response had a good overall survival rate that was twice that of no response. However, the study needs to recruit more case numbers and statistics with clinicopathological factors [133].

### 3.13. Dihydroxyphenylalanine (DOPA)

In addition to the well-known carboxylate metabolism, there is fatty acid biosynthesis and amino acid biosynthesis. Neurotransmitters are considered an important aspect, involving signaling pathways in the brain, reflex actions, nerve networks, memory, emotions, etc. Normal and tumoral neuroendocrine cells can uptake and decarboxylate amine precursors (such as L-DOPA and 5-hydroxytryptophan) to produce biogenic amines, such as catecholamines and serotonin [134]. For neuroendocrine tumors (NET), fluorine-18-dihydroxyphenylalanin (^18^F-DOPA) has been engineered to capture signals because these tumors can accumulate and decarboxylate biogenic amines. Through activation of large amino acid transporters (LAT, SLC7A5/A8), ^18^F-DOPA is absorbed into tumors and further decarboxylated by DOPA decarboxylase to produce ^18^F-DOPAmine, which is then transported by vesicular monoamine transporters (VMAT, SLC18A1/A2) and trapped in cells [135]. ^18^F-DOPA has comprehensive applications in different neuroendocrine tumors, including medullary thyroid cancer (MTC), pancreatic NET, and paragangliomas/phaeochromocytomas [136]. Furthermore, ^18^F-DOPA can differentiate between Parkinson’s disease and dopaminergic transmission disorder [137]. ^18^F-DOPA enables presynaptic dopaminergic function to be quantified, and specific regions of linear reduction in ^18^F-DOPA uptake in idiopathic Parkinson’s disease to be observed [137].

In addition to ^18^F-DOPA, gallium-68-somatostatin analogs (DOTA-NOC, DOTA-TOC or DOTA-TATE) have also shown high diagnostic NET imaging on PET/CT scans targeting the accuracy of the somatostatin receptor (SSR). Several studies have compared the uptake and additional functional activity of SSR PET/CT and ^18^F-DOPA PET/CT. Although patients with gastroenteropancreatic and thoracic NETs may prefer SSR PET/CT as a priority strategy, ^18^F-DOPA PET/CT remains another viable option [138].

### 3.14. Glucosamine

Glycosylation is a hallmark of various neurological disorders. A current study found that N-linked protein glycosylation in the brain led to glucosamine metabolism through glycogen catabolism [139]. Brain glycogen contains 25% glucosamine, and the mass spectrometry imaging method can reveal the distribution of brain glycogen [139]. Additionally, glycogen is a branched glucose polymer that is synthesized from the activated glucose donor, uridine diphosphoglucose (UDP) [139]. In some cancer cells, we can observe that the cell is deficient in energy stores that require glucose uptake. There is also evidence that glycogen is more than a glucose cache, and is a critical storage macromolecule for the brain protein N-glycosylation, which impacts myriad subsequent cellular processes. Therefore, a fluorescent tool labeled fluorine-18 has been developed to detect glycogen [140].

Returning to the metabolic background, the glucosamine analog, N- (methyl-2-fluoroethyl) -1H- [12,3] triazole-4-yl) glucosamine (NFTG) has been applied to some animal models. Witney’s group confirmed that some cancer cells do not have high glucose uptake, but have strong glucose storage and glycogen (glycogenesis). Annotation of glycogenogenesis, glycogen, and glycogen synthase 1 expression in cancer cells with ^18^F-NFTG is advantageous in understanding the potential ability of ^18^F-NFTG to specifically image certain quiescent cells (G_0_–G_1_ phase). Furthermore, ^18^F-NFTG provides higher-specificity tumor imaging under inflammatory conditions than ^18^F-FDG [141].

## 4. Hypoxia-Targeted Tracers

As discussed in the above-mentioned chapters, cancer cells adapt rapidly to changes in oxygen pressure by converting metabolic intermediates into one another. It has also been demonstrated that the hypoxia environment can modify cancer cells’ use of metabolites, modify their acid-base environment, and communicate with the microenvironment in a way that promotes cell malignancy and resistance to treatment [142,143]. As a result, many compounds have been designed to mark hypoxia for the purpose of diagnosis.

### 4.1. Diacetyl-bis (N4-Methylthiosemicarbazone)

For hypoxic tumors, angiogenesis and metabolite uptake rates differ from those of normal tissues. After careful classification, there are different characteristics, and one of these is that there is a high degree of angiogenesis, with cells increasing the efficiency of proliferation and necrosis. This can be shown from ^18^-FDG PET. The other is an area of insufficient angiogenesis, lack of oxygen and metabolites, and therefore cell cycle arrest, rather than necrosis. For this feature, copper-diacetyl-bis(*N*^4^-methylthiosemicarbazone) (Cu-ATSM) has better imaging quality. Cu-ATSM is a neutral and lipophilic copper(II)bis(thiosemicarbazone) that has shown rapid diffusion into cells and has been shown in vitro to be highly selective for hypoxic tissues. Positron-emitting copper isotopes with high specific activity can be produced on a biomedical cyclotron. These isotopes are ^60^Cu (t1/2, 23.7 min; b1, 93%; electron capture [EC], 7%), ^61^Cu (t1/2, 3.33 h; b1, 61%; EC, 39%), and ^64^Cu (t1/2, 12.7 h; b1, 17.4%; EC, 43%). The availability of these copper radionuclides allowed us to conduct this investigation to confirm the hypoxic selectivity of Cu-ATSM in vivo in an animal tumor model [144].

In a study by Yoshii et al., ^64^Cu-ATSM accumulated in the abundant region of CD133^+^ cells with cancer stem cells (CSCs) characteristics. These results are also consistent with the fact that Cu-ATSM can reveal certain subsets in which the cell cycle is arrested, quiescent, and poorly angiogenic [145]. Furthermore, the Hino-Shishikura group combined ^62^Cu-ATSM PET/CT imaging to dissect GBM and primary central nervous system lymphoma [146].

#### 2-nitroimidazole

Unlike metabolic intermediates or biosynthetic products, nitroimidazole is an organic compound that cannot be synthesized in the body [147]. Flumiisonidazole (^18^F-FMISO) is designed to be used as a radiopharmaceutical for PET imaging hypoxia. When ^18^F-FMISO enters living cells, the nitro group of FMISO is reduced. In nonhypoxic cells, reduced FMISO molecules can be oxidized to diffuse into the free extracellular circulation and ultimately excreted [148,149,150]. However, in hypoxic tumor cells, this oxidation does not occur and the FMISO molecules accumulate. Therefore, the anoxic and nonanoxic regions are separated. This may reflect local acidity and hypoxia in imaging systems due to the location and high turnover metabolism of certain types of Warburg effect tumors [151].

Several small-scale imaging trials using ^18^F-MISO have shown that hypoxia, as assessed by PET, may be associated with overall survival and local control in patients with head and neck cancer [152]. There are also studies on HER2^+^ breast tumors [153] and brain tumors [154]. However, the actual clinical contribution has not been clearly established. For this, the specificity and sensitivity of ^18^F-MISO will have to be further improved and a better understanding reached regarding exactly how hypoxia levels affect treatment planning decisions. In the future, it is expected that ^18^F-MISO will be used for hypoxia imaging to screen patients before prescribing hypoxia-guided medications. It can also be used as a post-treatment effectiveness measure for radiation therapy and chemotherapy [150,155]. Additionally, the application of ^18^F-MISO in cardiac hypoxia imaging is limited because of the low contrast between the target and background and the long injection-to-imaging delay that requires high injection activity [156]. 

### 4.2. Azomycin Arabinoside

The application of azomycin arabinoside is similar to that of 2-nitroimidazole, which is labeled with fluorine and used for PET. In a phase 1b/II translational study, the ^18^F-AZA has been recruited to evaluate the hypoxia in soft-tissue sarcoma [157]. In this study, it was also reported that ^18^F-AZA had potential advantages over ^123^I-IAZA because of its higher resolution, higher contrast, and better radiation dosimetry, and higher TBR ratios than ^18^F-MISO [158]. Higher clearance of ^18^F-AZA compared to 18F-MISO decreased specific background activity and thus provided better lesion contrast for PET [159]. Additionally, a clinical trial is enrolling 20 participants in order to evaluate tumor hypoxia in all-cell non-small lung cancer using ^18^F-AZA PET prior to radiotherapy (NCT02701699). 

### 4.3. Future Prospects

A molecular imaging approach can facilitate rapid and noninvasive identification of patients who are not responding to therapy. This allows a change in treatment approach to be made at a very early stage. It reduces side effects for patients, as well as providing an early indication of whether a treatment is effective. As diagnostic markers, differences in the progression of metabolic abnormalities at early and late stages have been observed. Therefore, metabolic pathway-based detection strategies are essential. Incorporating metabolism-related radiotracers is one of the possible approaches to visualization. The most significant benefit of tracking metabolic signatures in disease and cancer will come from understanding metabolic changes, although there are some limitations (Table 1). Metabolism-related radiotracers are based on metabolites that have a low risk of adverse effects in clinical practice. Furthermore, the performance and efficiency of the metabolic enzymes involved can be considered as additional parameters for improving sensitivity and accuracy. Metabolism reprogramming events and metabolomic profiling of diseases and cancer are still under investigation. It is expected that more metabolites will be identified and used for enhancing noninvasive imaging systems by developing and improving radiotracers.

## Figures and Tables

**Table 1:** Selected radiotracers of cellular metabolism for imaging approaches.

Tracer	Metabolites	Major Applications	Radiolabeling	Functions
Fluorodeoxyglucose (FDG)	Glucose analogue	Multiple cancers	^18^F	Carbohydrate metabolism
Pyruvate	Pyruvate	Multiple cancers	^11^C	Carbohydrate metabolism
Fluorodeoxygalactose (FDGal)	Galactose analogue	HCC	^18^F	Carbohydrate metabolism
Choline	Choline	HCC, prostate, parathyroid, cholangiocarcinoma	^11^C, ^18^F	Phospholipid metabolism
Acetate	Acetate	Prostate cancer	^11^C	Lipid biosynthesis
Fluoro-2,2-dimethylpropionic acid (FPIA)	Carboxylic acid	Solid tumor	^18^F	Lipid biosynthesis
Fluciclovine (FACBC)	L-leucine analogue	Prostate, myeloma	^18^F	Amino acid biosynthesis
Methionine (MET)	Methionine	Glioma, germinoma, myeloma, oral	^11^C	Amino acid biosynthesis
Glutamine	Glutamine	Paraganglioma, pheochromocytoma, metastatic renal, breast, glioma.etc	^11^C, (^18^F) *	Amino acid biosynthesis
Fluoropropyl-L-glutamic acid (FSPG)	Glutamate analogue	Head and neck, colorectal, non-Hodgkin lymphoma	^18^F	Amino acid biosynthesis
Fluoroethylthyrosine (FET)	L-tyrosine	GBM, gliomas	^18^F	Amino acid biosynthesis
Fluorothymidine (FLT)	Thymidine	Pancreases, Gliomas, NSCLC	^11^C, ^18^F	Nucleotide biosynthesis
Dihydroxyphenylalanine (DOPA)	Phenylalanine, precursor of dopamine	Neuroendocrine tumor, parkinsonian syndromes	^18^F	Neurotransmitter metabolism
N-(methyl-(2-fluoroethyl)-1H-[1,2,3]triazole-4-yl)glucosamine (NFTG)	Glucosamine analogue	Quiescent cells	^18^F	Glycogen metabolism
Fluromisonidazole (FMISO)	Nitroimidazole analogue	Head and neck, breast, brain, cardiac hypoxia imaging	^18^F	Hypoxia status
Luoroazomycin arabinoside (FAZA)	Nitroimidazole analogue	Lung, Sarcoma	^18^F	Hypoxia status
Diacetyl-bis (N^4^-methylthiosemicarbazone) (ATSM)	Copper	colon carcinoma, brain	^62^Cu, ^64^Cu	Hypoxia status (Commonly used hypoxia sensors but not for metabolic applications)

* ^18^F-labeled (2S, 4R)-4-fluoro-I-glutamine (4F-GLN).

## Data Availability

Not applicable.
